# Oxygen Gas as a Remedy in Disease

**Published:** 1870-10

**Authors:** 


Oxygen Gas as a Remedy in Disease.—In the May number of this journal, we published entire the first three chapters of the able essay of Dr. A. H. Smith on this subject. The four remaining chapters treat directly of the therapeutic uses of oxygen. We summarize this portion of the paper as follows:

The diseases in which oxygen is most useful are of two classes: those in which respiration is at fault, mostly acute; and those in which there is defective nutrition or excretion, chiefly chronic.

*Oxygen in Impaired Respiration.*—Every condition in which there is dyspnœa will be benefited—temporarily at least—by the use of oxygen. The essence of dyspnœa is a disproportion between the quantity of venous blood in the lungs, and the amount of oxygen in the air cells; by breathing pure oxygen the equilibrium is restored.

*Asthma.*—The dyspnœa in this affection would at once suggest the use of oxygen. Beddoes used it in twenty-two cases, ten of which were cured, nine relieved, and three received no benefit.

Demarquay reports a case cured and one greatly relieved, and Birch reports one of absolute cure.

In the hands of Dr. H. Pinkney, in one case it relieved the paroxysms and reduced their frequency. Beigel reports three cases, one hereditary, in which oxygen was inhaled with liq pot. arsen. The paroxysms were relieved, and the tendency to recur decreased.

Surg. Gen. Barnes gave oxygen to the late Secretary Stanton, who had severe paroxysms of asthma during his last illness. It controlled the asthma completely.

Dr. Smith himself has given oxygen in two cases of asthmatic paroxysms, with the effect of immediate relief, but it was not continued during the intervals.

*Pulmonary Emphyesma.*—Dr. Beigel reports a very severe case in which, by inhalation of one gallon of oxygen every three hours, there was a prompt amelioration of the distressing symptoms; at the end of six weeks they had nearly all disappeared. During this time there was a solution of common salt, repeatedly taken by inhalation [atomized], also pure water. Subsequent to this a solution of tannin and morphia was used in connection with the oxygen. At the end of two months, the patient returned to business, still obliged to use the oxygen at intervals. He continued to improve under its use.

Another case is reported where oxygen was given only for a short time, and mainly for dyspnœa; this was relieved at every administration; the countenence lost its livid hue, the pulse fell from 122 to 100, and the respiration from 36 to 20.

*Croup.*—Dr. Beigel describes a severe case of croup in which the usual modes of treatment had proved unavailing, the respiration being 40 and noisy, and the pulse too rapid to be counted, lips livid and face pale. The inhalation of one cubic foot of oxygen was followed by decided improvement: it was repeated, and the child made a prompt recovery. Dr. Miguel reports a case with symptoms identical with the above, in which recovery followed the use of oxygen. "I have been called in to give oxygen in several cases of croup, but always in the last stages, and when dyspnœa had been long continued. Under these circumstances, although the dyspnœa has been relieved, death has taken place."

Oxygen will do in croup all that can be done by tracheotomy, but neither is competent to undo the mischief wrought by prolonged dyspnœa. Hence the rule, with oxygen as with the knife—*use it early.*

*Diphtheria.*—Dr. Beigel reports two cases in which a state of coma disappeared at once on the administration of oxygen; the eyes became bright, the pulse rose, the face became ruddy, and the appetite soon returned.

I have given this agent in only a single case of diphtheria; the patient was in *articulo mortis,* but life was sustained by it for five hours, the patient choking at every suspension of it, and dying in five minutes after its administration was stopped. In this disease we have, added to the dyspnœa, a septic condition of the blood, which greatly depresses the powers of life.

*Pneumonia.*—Dr. Golden reports a case of double pneumonia, with great dyspnœa, which resolved in four days under the use of oxygen.

Despite its contra-indication in acute inflammation, "I should not hesitate to use it in a case of double pneumonia;" the fear that it will excite pneumonitis by its local action is without foundation.

*Capillary Bronchitis.*—In this affection oxygen is especially valuable, as, while relieving the dyspnœa, it diminishes the formation of mucus in the lungs, which latter is largely the result of labored repiration. Inhalation of oxygen removes the necessity for forced breathing, and thus lessens congestion of the mucous membrane, and lessens the tendency to effusion. Hence it is not only palliative but curative.

In *opium poisoning* and in *cholera* collapse there exist rational reasons, and no little evidence, practically, in favor of the use of oxygen.

*The Use of Oxygen in Diseases with Defective Nutrition.*

*Phthisis.*—We have abundant evidence to show that in this disease, as in others in which nutrition is defective, as a rule, the use of the gas favors assimilation, and results in a gain in weight. But whether it exerts an influence on the morbific principle, the essence of the disease, facts alone can determine, and, up to the present time, sufficient facts have not been gathered to warrant an answer.

There is considerable evidence to show that oxygen may arrest the progress of the disease for a time, and possibly eradicate it. Experiments show that oxygen, in contact with a wound or ulcer, acts as a stimulant, and, carried too far, sets up active inflammation. Herein we have a therapeutical indication, and possibly a solution of our failures with oxygen in phthisis, especially in its advanced stages. Too little diluted it may excite inflammatory action in the pulmonary ulcers, and thus aggravate the general disease. There are cases on record, with cavities and purulent expectoration, in which oxygen has been for a time beneficial, but has then produced symptoms of local inflammation. By diminishing the action in time, this effect would be avoided, and the benefit gained be retained.

Demarquay reports two cases and quotes a third in which phthisis had advanced so far as to develop cavities, and extreme emaciation and prostration, and when oxygen was given—in small quantities at first, and carefully increased—with the effect to bring the patients up to a good degree of vigor and comfort, but not radical cure.

M. Hervé de Lavour, also quoted by Demarquay, has given oxygen in nine cases of this disease. In three cases, under the use of four gallons daily to begin with, increased to twelve gallons taken at two sitings, the patients made rapid and sure improvement. The other six cases received little or no substantial benefit from the treatment.

Birch reports two undoubted cases of absolute cure of phthisis by oxygen.

Experiments made upon ten consumptives in the New York Hospital, giving each six gallons of oxygen daily, caused the weight of six to increase in four weeks an aggregate of forty-nine and a-half pounds, while the other four lost seven pounds. In several cases, there was at first a rise in temperature, which disappeared on suspending, for a short time, the gas.

After relating three cases of his own, which seem to show perfect cures, and statement of several in which success was less flattering, Dr. S. says: " While not prepared to endorse the opinion of Birch, that, with the use of oxygen, the cure of consumption in its earlier stages should be the rule rather than the exception, I have no hesitation in saying, that I have more confidence in it than in any and all other remedies."

In *anœmia*, oxygen is strongly recommended, and two cases are detailed to show its almost magical effect in this condition.

In *dyspepsia* it has succeeded when all other means failed. Trousseau commends it in highest terms, and speaks of having "*recalled to life* women regarded as lost from great depression of the digestive powers. Demarquay relates a similar experience. Birch insists on the power of oxygen to remove congestions of the liver, and to relieve the dyspepsias resulting therefrom.

In *diabetes,* while there are no records of cure by oxygen, there are several cases which have received from it decided benefit.

La Passe, Hill, Demarquay, Birch, and Dr. Mackey all testify to the power of oxygen to promptly relieve the ordinary headaches, neuralgic and "nervo-congestive."

Of cases of *epilepsy* treated with oxygen by various observers, four were cured, while five received no benefit.

*Application of Oxygen in Surgery.*

Demarquay compelled a dog, with a wound near the axilla, which looked dull and had grayish edges, to breathe pure oxygen. In two minutes the wound became rose red. The surface presented some small ecchymotic spots, which bled slightly, and an abundant serous discharge flowed from the surface of the wound. On suspending the inhalation, the wound changed to its former color at once, and the surface became dry and dull-looking, like a mirror breathed upon. He observed a like effect from the same course on the human subject. Taking the hint from such experiments, oxygen has been used in many cases of slow-healing wounds and sores. Many cases are reported of ulcers, scrofulous and syphilitic, of long standing, which have been most happily cured by this means. One of these ulcers had resisted all treatment for 18 years; six months after cure it had not reappeared.

